# Inflammatory markers as predictors of antidepressant response: a precision-psychiatry synthesis

**DOI:** 10.1192/j.eurpsy.2026.10927

**Published:** 2026-07-31

**Authors:** B. Mihaela, D. Jelaga

**Affiliations:** Department of Mental Health, Medical Psychology and Psychotherapy, Nicolae Testemițanu State University of Medicine and Pharmacy, Chisinau, Moldova, Republic of

## Abstract

**Introduction:**

Low-grade systemic inflammation may contribute to heterogeneity in antidepressant outcomes. Peripheral biomarkers could inform first-line choice, yet the predictive value of C-reactive protein (CRP), interleukin-6 (IL-6) and neutrophil-to-lymphocyte ratio (NLR) remains uncertain.

**Objectives:**

(1) To quantify associations between baseline CRP/IL-6/NLR and 6–12-week response/remission in adults with major depressive disorder (MDD); (2) to test class-specific signals (SSRIs vs SNRIs) and pragmatic cut-offs; (3) to explore moderators (age, sex).

**Methods:**

Systematic searches of MEDLINE/PubMed, Embase, PsycINFO and Web of Science (2020–2025) identified adult MDD cohorts initiating antidepressants with pre-treatment biomarkers and 6–12-week outcomes (HAM-D/MADRS/CGI). Included: observational/longitudinal cohorts and meta-analyses; excluded: ECT/rTMS, adolescent-only or psychotic-majority samples, and studies without therapeutic outcomes. Records: 786; deduped: 598; full texts: 23; included: 7 (6 primary cohorts + 1 meta-analysis); excluded: 16 (non-pharmacological=6; irrelevant outcomes=5; ineligible populations=3; no baseline biomarkers=2). Populations: ≈2,291 after overlap checks; age 18–72 years; women 55–70%; mixed outpatient/inpatient. Primary outcomes: response (≥50% HAM-D/MADRS) and remission (e.g., HAM-D≤7/MADRS≤10) at 6–12 weeks. Units were harmonised (CRP mg/L; IL-6 pg/mL). Adverse events and adherence were not consistently reported.

**Results:**

CRP: in a prospective cohort (n=1,024), hs-CRP below a data-driven threshold (~0.61 mg/L) was associated with higher 12-week remission overall (OR=2.16–2.28). In real-world data (n=918), 77.2% had CRP<1 mg/L and 22.8% ≥1 mg/L; a class-by-CRP interaction emerged: SNRIs outperformed SSRIs at CRP≥1 mg/L (HR=1.652; 95%CI 1.031–2.654), whereas outcomes were better with SSRIs at CRP<1 mg/L than in high-CRP patients (HR=1.257; 1.003–1.574).

IL-6: in the 1,024-patient cohort, IL-6<1.45 pg/mL predicted 12-week remission with escitalopram (OR=1.72; 95%CI 1.17–2.51). In an independent paroxetine cohort (n=104), lower baseline IL-6 independently predicted greater 8-week HAM-D reduction.

NLR: one eligible inpatient study (n=124; 64.5% women) found higher baseline NLR associated with poorer antidepressant response in women; evidence is inpatient-skewed, thresholds unvalidated, and replication lacking. No biomarker-related adverse events were reported.

**Conclusions:**

Across evidence, low inflammation (CRP<~1 mg/L and/or low IL-6) is associated with higher 6–12-week remission on SSRIs; at CRP≥1 mg/L, SNRIs show relative advantage. NLR shows limited, sex-dependent signals and is not ready for treatment selection. A minimal pre-treatment panel (hs-CRP ± IL-6) appears feasible to guide SSRI vs SNRI choice. Major limitation: observational designs with assay heterogeneity; reporting of adverse events and adherence was inconsistent; biomarker-guided randomised trials are needed.

**Disclosure of Interest:**

None Declared

